# Unlocking the Potential of Freeze‐Dried Broccoli Powder: A Novel Approach to Enhancing Cognitive Resilience in Temporal Lobe Epilepsy

**DOI:** 10.1002/fsn3.70079

**Published:** 2025-03-08

**Authors:** Yanan Gong, Hongzhen Zhou, Xinle She, Yan Guo, Yongzhong Zhou, Nan Peng, Guoli Zhou, Tengwei Gao, Furong Liu, Yiqian Wang, Jing Ye, Jing Jin, Rui Zhang

**Affiliations:** ^1^ Ningxia Key Laboratory of Craniocerebral Diseases, Incubation Base of National Key Laboratory Ningxia Medical University Yinchuan China; ^2^ School of Basic Medical Science Ningxia Medical University Yinchuan China; ^3^ School of Public Health and Management Ningxia Medical University Yinchuan China; ^4^ Key Laboratory of Environmental Factors and Chronic Disease Control Ningxia Medical University Yinchuan China; ^5^ Department of Medical Records General Hospital of Ningxia Medical University Yinchuan China

**Keywords:** cognitive function, freeze‐dried broccoli powder, oxidative stress, temporal lobe epilepsy

## Abstract

Cognitive impairment frequently accompanies temporal lobe epilepsy (TLE), which is linked to oxidative stress. Freeze‐dried broccoli powder (BROC) exhibits diverse biological activities, particularly its antioxidant properties. Our study explored the effects of BROC on post‐epileptic cognitive function. Male mice with lithium‐pilocarpine‐induced epilepsy were randomly divided into three groups: control group, model group (basal diet), and broccoli intervention group (basal diet supplemented with 4% BROC). Following 60 days of intervention, cognitive function was evaluated using IntelliCage Learning Tests, Novel Object Recognition, and Shuttle Box Tests. The hippocampal levels of malondialdehyde (MDA) and antioxidant enzyme activities were measured, and the protein expression levels of HO‐1, NQO1, and Nrf2 were analyzed by Western blot. The results demonstrated that broccoli intervention significantly ameliorated cognitive deficits in epileptic mice, decreased hippocampal MDA levels while enhancing antioxidant enzyme activities, and upregulated the expression of HO‐1, NQO1, and Nrf2. Consequently, BROC effectively attenuates OS and ameliorates cognitive deficits in TLE mice, suggesting its potential therapeutic value for post‐epileptic cognitive dysfunction.

## Graphical abstract

The graphic abstract presents a focused study on the neuroprotective role of freeze‐dried broccoli powder in epilepsy, centering on the activation of the Nrf2 pathway. This activation leads to enhanced antioxidative enzyme expression (GSH‐Px, HO‐1, NQO1) and decreased OS markers (MDA), suggesting a mechanistic pathway for cognitive function improvement in epilepsy models. The investigation highlights the therapeutic efficacy of broccoli‐derived compounds in addressing cognitive impairments, offering a novel, adjunctive approach to epilepsy management.

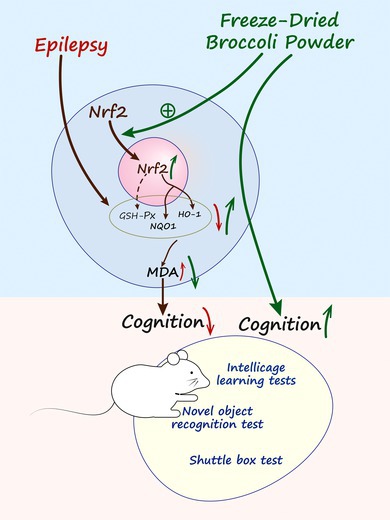

**Abbreviations**
ARE: Antioxidant Response Elements
BROC: Freeze‐dried broccoli powder
CAT: Catalase
CHRM1: Cholinergic Receptor, Muscarinic 1
EP: Epilepsy
GSH‐Px: Glutathione Peroxidase
GSTm1: Glutathione S‐transferase M1
HO‐1: Heme Oxygenase 1
MDA: Malondialdehyde
NQO1: Quinone Oxidoreductase 1
Nrf2: Nuclear factor erythroid‐2‐related factor 2
OS: Oxidative Stress
ROS: Reactive Oxygen Species
SFN: Sulforaphane
SOD: Superoxide Dismutase
T‐AOC: Total Antioxidant Capacity

## 1 Introduction

Temporal lobe epilepsy (TLE), representing one of the most prevalent forms of epilepsy, is predominantly characterized by pharmacoresistance and is strongly associated with severe cognitive dysfunction (Phuong et al. 2021). Cognitive functions, including executive, learning, and memory abilities, are fundamental to quality of life. Although current antiepileptic medications demonstrate efficacy in seizure control, they exhibit limited therapeutic benefit in addressing the cognitive deficits associated with the disorder (Postnikova et al. 2019; Rodriguez‐Cruces et al. 2022).

The lithium‐pilocarpine‐induced epilepsy model in mice represents a well‐established experimental paradigm that effectively replicates human status epilepticus. This model is distinguished by its accurate reproduction of multiple pathological features, including spontaneous recurrent seizures, behavioral alterations, electroencephalographic irregularities, and neuropathological manifestations. Importantly, it reflects the cognitive deficits that are frequently seen in chronic epilepsy. Although many epilepsy patients can manage their seizures with pharmaceuticals, the capacity of these medications to improve cognitive functions remains unclear. This limitation emphasizes the imperative need for investigating and developing therapeutic strategies that offer both enhanced safety profiles and improved efficacy in addressing cognitive deficits and alleviating patient burden (Ahmed Juvale and Che Has 2020).

Oxidative stress (OS) represents a persistent pathophysiological challenge throughout the development of epilepsy. Excessive reactive oxygen species (ROS) in the body can induce oxidative stress, leading to potential neuronal damage and cognitive impairment (Teleanu et al. 2022). Malondialdehyde (MDA) and glutathione peroxidase (GSH‐Px) are widely recognized as markers of oxidative stress. In the state of oxidative stress, the levels of antioxidant proteins such as quinone oxidoreductase 1 (NQO1), heme oxygenase 1 (HO‐1), glutathione peroxidase 1 (GSH‐Px1), and glutathione S‐transferase M1 (GSTm1) exhibit significant downregulation, while the level of MDA tends to increase (Detcheverry et al. 2023; Mohideen et al. 2023). Nuclear factor erythroid 2‐related factor 2 (Nrf2) is an essential intrinsic antioxidant factor that plays a critical role in protecting against oxidative damage through the Nrf2 antioxidant response element (ARE) signaling pathway (W. Wang et al. 2013). The reduction in Nrf2 expression can further aggravate neuroinflammation and apoptosis (Bhowmick et al. 2019). Therefore, maintenance of redox homeostasis emerges as a critical therapeutic target for mitigating epilepsy‐associated neuroinflammation.

Broccoli, a cruciferous vegetable, contains abundant phytochemicals, including phenolic compounds, organic sulfides, carotenoids, minerals, and vitamins (Ramirez et al. 2020). Research has elucidated that different processing methods markedly affect the concentration of bioactive substances in broccoli. Notably, freeze‐dried broccoli exhibits superior nutritional composition and antioxidant activity retention compared to conventional air‐dried preparations (Kim et al. 2021; Mohammadi et al. 2020; Sharma et al. 2011). These bioactive constituents confer multiple therapeutic properties, including antioxidant, anti‐inflammation, anti‐carcinogenic, and cytoprotective properties (Mandrich and Caputo 2020; Raiola et al. 2017). Additionally, accumulating evidence indicates that broccoli‐derived compounds, particularly sulforaphane, activate the Nrf2‐ARE signaling pathway, thereby upregulating the levels of antioxidant enzymes like GSH‐Px, catalase (CAT), and superoxide dismutase (SOD) ultimately enhancing cellular antioxidant defense mechanisms (Ruhee and Suzuki 2020; Zhang et al. 2023a). However, the therapeutic potential of broccoli in ameliorating cognitive impairment in lithium‐pilocarpine‐induced epileptic mice and its antioxidant effects on hippocampal tissue remain to be fully elucidated. Therefore, our aim is to investigate whether freeze‐dried broccoli powder (BROC) can attenuate cognitive and learning memory deficits, enhance hippocampal oxidative resistance, and exert neuroprotective effects in an experimental epilepsy model.

## 2 Materials and Methods

### 2.1 Reagent

Freeze‐dried broccoli powder, hairy root culture of *Physalis angulata* containing solasodine (purity: ≥ 98%) and atropine (purity: ≥ 98.0%) were sourced from Sigma‐Aldrich Corporation (St. Louis, Missouri); lithium chloride was procured from Tianjin Haicheng Chemical Co. Ltd. (Tianjin, China); MDA, GSH‐Px, CAT, SOD, and Total Antioxidant Capacity (T‐AOC) assay kits were acquired from Nanjing Jiancheng Bioengineering Research Institute; the total protein extraction kit and nuclear protein extraction kit were obtained from Nanjing KeyGen Biotech Co. Ltd. All other reagents, ensured to be of the highest purity, were purchased from various sources (Tables 1, 2, 3, 4).

**TABLE 1:** Basic feed and its component analysis.

Nutritional content	Composition content (g/kg)
Moisture	≦ 100
Crude Protein	≧ 180
Crude Fat	≧ 40
Crude Fiber	≦ 50
Crude Ash	≦ 80
Calcium	10–18
Total Phosphorus	6–12

**TABLE 2:** Amino acid nutritional content and its concentration.

Nutritional content	Composition content (g/kg)
Lysine	13.5
Methionine + Cysteine	7.6
Arginine	11.9
Histidine	6.0
Tryptophan	3.6
Phenylalanine + Tyrosine	14.9
Threonine	8.7
Leucine	14.5
Isoleucine	9.5
Valine	10.5

**TABLE 3:** Mineral components and their concentrations.

Nutritional content	Composition content (g/kg)
Magnesium	2.27
Potassium	6.53
Sodium	3.11
Iron	195
Manganese	105
Copper	14.1
Zinc	55
Iodine	0.55
Selenium	0.12

**TABLE 4:** Vitamin components and their concentrations.

Nutritional content	Composition content (IU/kg, mg/kg)
Vitamin A	13,692
Vitamin D	1339
Vitamin E	115.4
Vitamin K	5.5
Vitamin B1	15
Vitamin B2	17.4
Vitamin B6	10
Vitamin B12	0.035
Niacin	74.9
Pantothenic acid	32.7
Biotin	0.174
Choline	1360
Folic acid	7.50

### 2.2 Diet Composition and Preparation of Freeze‐Dried Broccoli Powder

The basal diet consisted of standard ingredients, including corn from Northeast China, wheat, imported fish meal, chicken meal, soybean meal, soybean oil, sodium chloride, limestone, calcium hydrogen phosphate, choline chloride, methionine, vitamin A, vitamin D3, dl‐α‐tocopheryl acetate, menadione sodium bisulfite, thiamine nitrate, riboflavin, pyridoxine hydrochloride, cyanocobalamin, nicotinamide, calcium D‐pantothenate, folic acid, D‐biotin, basic copper chloride, ferrous sulfate, manganese sulfate, zinc hydroxymethionine chelate, and selenium yeast (composition detailed in Tables 1, 2, 3, 4).

Broccoli was harvested at day 12 of growth (Lin et al. 2022; Mahn et al. 2022). After washing and cutting it into pieces, the broccoli was frozen at −24°C for 10 h, followed by freeze‐drying and grinding it into powder. The freeze‐dried broccoli powder is rich in various bioactive compounds, including carotenoids, vitamins, folic acid, and proteins.

### 2.3 Animals and Treatment

Eight‐week‐old healthy C57BL/6J mice were obtained from the Laboratory Animal Center of Ningxia Medical University, assigned IACUC animal code SCXK(Ning) 2017–0001. The mice were housed in a controlled environment with a 12 h light–dark cycle, with a constant temperature of 22°C ± 2°C and humidity of 55% ± 15%. They had ad libitum access to food and water. The experimental procedures were approved by the Animal Care and Use Committee of Ningxia Medical University (Approval No. NXMU2020‐021) in accordance with the guidelines of the National Institutes of Health's Guide for the Care and Use of Laboratory Animals. All efforts were made to minimize pain and reduce the number of animals used in the study.

Male mice were randomly assigned to one of three groups: a control group, an epilepsy group (EP), and a broccoli intervention group (EP + BROC) (*n* = 10). The control and epilepsy groups received a basal diet. In contrast, the broccoli intervention group was administered the basal diet enriched with an additional 4% freeze‐dried broccoli powder (BROC) for a total intervention period of 88 days.

**FIGURE 1:**
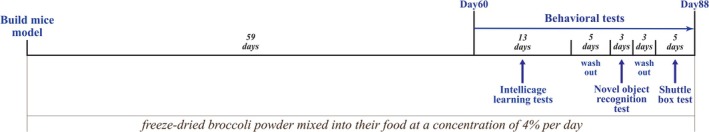
Experimental timeline.

At the beginning of the experiment, both the epilepsy and broccoli intervention groups underwent temporal lobe seizure induction, while the control group received saline. Induction of lithium‐pilocarpine‐induced TLE consisted of administering lithium chloride (130 mg/kg body weight, i.p.) 18 h prior to the pilocarpine injection. To minimize complications during the acute seizure phase, atropine (1 mg/kg body weight, i.p.) was administered 30 min before the pilocarpine injection to reduce seizure‐associated peripheral cholinergic side effects. Mice were weighed once a week, and behavioral tests were conducted on day 60 post‐treatment. The experimental timeline is indicated in Figure 1. Following this, the mice were sacrificed 5 days after the final behavioral test for the assessment of the brain‐to‐body weight ratio calculation, enzymatic assay determination, and Western Blot analysis.

### 2.4 Behavioral Tests

The mice behavioral tests were conducted in an isolated, temperature‐controlled room to minimize stress to the animals. For the novel object recognition and shuttle box experiments, a video camera was strategically placed over the central area of the experimental setup and connected directly to a computer.

#### 2.4.1 IntelliCage Learning Tests

The IntelliCage system (TSE Systems GmbH, Germany) is composed of four rooms situated at the corners. Each room is equipped with two water bottles and an antenna for the card reader, which detects the microchips embedded in the mice as soon as they enter the room. To facilitate nose pokes, the infrared beam‐break sensors were programmed to either be active or inactive. Microchips were implanted subcutaneously in the nape of each mouse to facilitate data separation for individual identification. The experimental procedure was outlined as follows:

Free Exploration: Mice were allocated 1 day to explore the environment freely and to familiarize themselves with it. All doors remained open, and all water bottles were made accessible. The frequency of visits to each corner and the amount of water consumed by each mouse were carefully documented.

Nose‐poke learning: Throughout 6 days, all doors remained closed, and mice were compelled to explore the nose‐poke holes to obtain water. The number of visits to each corner by each mouse was meticulously recorded to ascertain corner preference.

Behavioral extinguishment: Mice were afforded a day of unrestricted exploration to extinguish any previously learned behaviors.

Positive Position Learning: Over a span of 6 days, the least preferred corner for each mouse was designated as the “correct” corner, whereas the remaining corners were deemed “incorrect.” Following the exploration of the nose poke holes, the doors were opened to permit water consumption. Both the number of correct visits and the volume of water consumed by each mouse were meticulously recorded to evaluate their capability in positive position learning.

Reversed position learning: Over a period of 6 days, the corner diagonally opposite to the previously designated “correct” corner was redefined as the new “correct” corner, whereas the remaining corners were designated as new “incorrect” corners. Subsequent to the exploration of the nose poke holes, the doors were opened to allow water consumption. The number of correct visits and the volume of water consumed by each mouse were meticulously recorded to assess their ability in negative position learning.

After completion of the IntelliCage Learning Test, all mice underwent a 5 days washout period.

#### 2.4.2 Novel Object Recognition Test

Following the IntelliCage Learning Test, the Novel Object Recognition (NOR) Test was implemented for mice. In the initial exploratory phase, the mice were exposed to two identical stimuli. During the discriminative assessment phase that followed, one of the familiar stimuli was replaced with an unfamiliar one to assess the recognition capabilities of the mice. The initial phase was initiated at 10:00 AM, with the evaluation phase beginning at 2:00 PM, thereby ensuring a 4 h interval between the two phases. We carefully recorded the amount of time spent examining each object, alongside the Distance in Zone–1 (old object), Distance in Zone–2 (novel object), Time in Zone (s)‐1 (old object), and Time in Zone (s)‐2 (novel object). The distance recognition index (%) is calculated as the distance traveled during the exploration of the novel object divided by the total distance traveled during the exploration of the novel and familiar objects. Similarly, the recognition index for time (%) is derived from the duration of interaction with the novel object over the total interaction time with both the novel and the familiar objects multiplied by 100. After completing the Novel Object Recognition Test, the mice were given a 3 days rest period.

#### 2.4.3 Shuttle Box Test

Following the completion of the Novel Object Recognition Test, we proceeded with the Shuttle Box Test. Initially, we defined the experimental parameters, which included the safety period (the interval between the presentation of the unconditioned stimulus and the conditioned stimulus), the duration of the conditioned stimulus (comprising either a high‐frequency stable sound or a flash of light), the duration and intensity of the unconditioned stimulus (electric shock), and the number of shuttle movements. In the memory acquisition phase, mice were initially placed in the left chamber, facing the end wall, and allowed to acclimate for a 5 min period before starting the trials. The Shuttle Box Test experiment video analysis system adhered to a specific procedure. Initially, a distinct conditioned stimulus was presented and sustained for a designated duration. If the mouse remained on the same side, an unconditioned stimulus was administered. Subsequently, following the receipt of an electric shock, the mouse migrated from the circular hole to the opposite side for a designated period, which was then followed by the presentation of conditioned and unconditioned stimuli on the opposite side. This process was repeated. During the training phase, each mouse engaged in dozens of trials a day for three consecutive days. All the mice received a day off, and the test phase was carried out on the fifth day. The total number of avoidance and escape responses, along with the average duration of these responses (in seconds), was diligently recorded.

### 2.5 Brain‐To‐Body Weight Ratio

After anesthetizing the mice, they were weighed, and their brains were quickly excised and rinsed with cold physiological saline. The ratio of brain‐to‐body weight was determined using the following formula:

$$\text{Brain}-\text{to}-\text{body weight ratio}=\text{Brain mass}\;\left(\mathrm{mg}\right)/\text{Weight}\;\left(g\right)$$

### 2.6 Enzymatic Assay Determination

To obtain the hippocampus samples from mice, the tissues were isolated, and a 10% tissue homogenate was prepared utilizing a tissue homogenizer. The supernatant was then meticulously collected through centrifugation for 5 min at 4°C and 12,000 rpm. For the assay, the test sample was combined with a solution of Coomassie Brilliant Blue G‐250 to induce the development of a color. After thorough mixing, the absorbance was measured at 595 nm using an ELISA reader to determine the protein concentration by comparison with a standard curve. The levels of malondialdehyde (MDA) and the activities of glutathione peroxidase (GSH‐Px), catalase (CAT), superoxide dismutase (SOD), and total antioxidant capacity (T‐AOC) in the hippocampus were quantified in accordance with the protocols provided by the respective assay kits.

#### 2.6.1 MDA Content

A reaction system was assembled using a 10% hippocampal tissue homogenate. This system was immersed in a water bath at 95°C for 40 min. Following incubation, the mixture was centrifuged at 3500–4000 rpm for 10 min. An enzyme‐linked immunosorbent assay (ELISA) analyzer was used to measure the absorbance of the resulting supernatant at 532 nm.

#### 2.6.2 GSH‐Px Activity

The enzyme reaction was incubated with 0.25% hippocampal tissue homogenate for 15 min. Absorbance was measured at a wavelength of 412 nm using an enzyme immunoassay analyzer.

#### 2.6.3 CAT Activity

A reaction system was established using a 10% (volume/volume) hippocampal tissue homogenate. Absorbance at 405 nm was quantified using an enzyme‐linked immunoassay analyzer.

OD (Optical Density) activity assessment involved diluting hippocampal tissue homogenate to a concentration of 0.25% and incubating it with the substrate at 37°C for 20 min. Absorbance was then measured at a wavelength of 450 nm using an enzyme immunoassay analyzer.

### 2.7 Determination of Protein Levels

The protein levels of HO‐1, NQO1, and Nrf2 were assessed using Western blot analysis. Proteins from both whole and nuclear extracts were isolated from mouse hippocampal tissue and standardized to a protein concentration of 5 μg/μL. The samples were then subjected to denaturation by heating in a water bath for 10 min. After denaturation, the proteins were separated via 10% SDS‐PAGE electrophoresis at a steady voltage of 100 V for 1.5 h, and subsequently transferred to a PVDF membrane in a transfer electrophoresis tank at a steady voltage of 25 V for 1.5 h with a current of 125 mA. To mitigate nonspecific interactions, the membrane was blocked with 5% skim milk in TBST at 4°C overnight. Incubation with the primary antibody was performed at 37°C for 2 h, followed by incubation at room temperature for 0.5 h with the secondary antibody. Protein bands were visualized using fluorescence detection, and images were subsequently analyzed.

### 2.8 Determination of mRNA Expression Levels

The mRNA levels of HO‐1 and NQO1 in total RNA were evaluated using real‐time quantitative PCR. Total RNA was extracted, and the concentration of RNA in each sample was standardized. Subsequently, the mRNA was reverse‐transcribed into cDNA with β‐actin serving as the internal reference standard, followed by PCR amplification. The amplification mixture consisted of 12.5 μL of TB Green Premix Ex Taq II, 1 μL of each forward and reverse primer, 9.5 μL of nuclease‐free water, and 1 μL of cDNA. The reaction conditions comprised an initial denaturation step at 95°C for 30 s, followed by 40 cycles of denaturation at 95°C for 15 s, annealing at 58°C for 30 s, and extension at 72°C for 30 s.

### 2.9 Statistical Analyses

Parametric data were reported as mean ± standard error of the mean (SEM). Statistical analyses were performed using SPSS 24.0 software for Windows (SPSS, Chicago, Illinois). A one‐way analysis of variance (ANOVA) followed by post hoc Student–Newman–Keuls (SNK) tests was utilized. Statistical significance was established at *p* < 0.05.

## 3 Results

### 3.1 Sign, Body Weight Gain, and Brain‐To‐Body Weight Ratio

Throughout the treatment period, no overt signs of toxicity were observed in the mice across all groups. As illustrated in Figure S1, there were no significant differences in body weight gain or brain‐to‐body weight ratio among the groups (*p* > 0.05).

### 3.2 Freeze‐Dried Broccoli Powder Ameliorated the Cognitive Dysfunction Induced by Epilepsy

**FIGURE 2:**
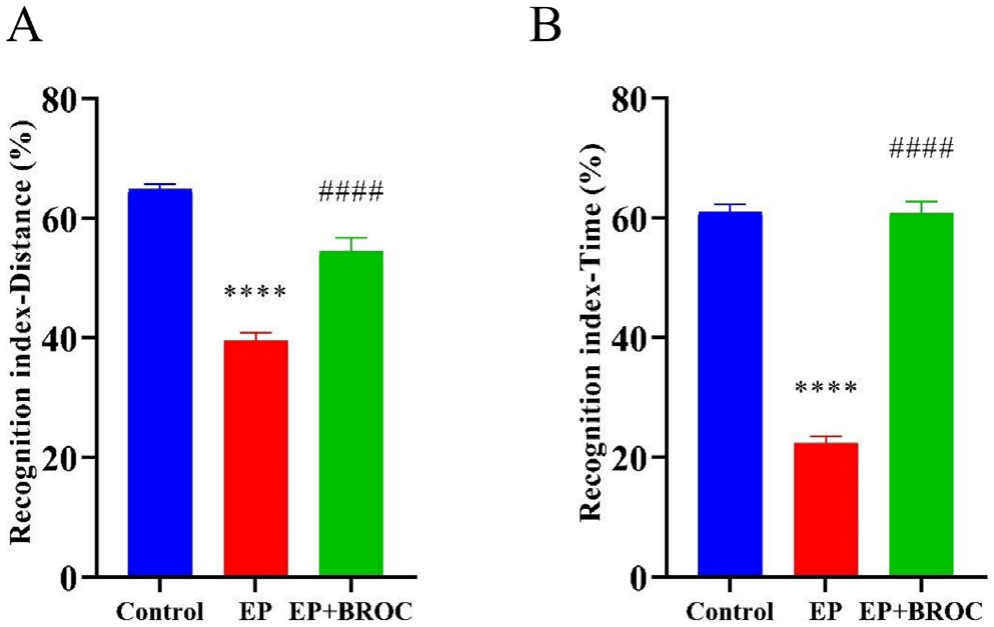
Analysis of mice behavior in novel object recognition test. (A) Recognition Index‐Distance, which was calculated based on distance spent on exploring the object. (B) Recognition Index‐Time, which was calculated based on time spent on exploring the object. (*n* = 10; mean ± SEM; One‐way ANOVA followed by SNK multiple comparison test; ^****^ *p* < 0.0001 vs. Control group, ^####^ *p* < 0.0001 vs. EP group).

Given the prevalence of cognitive impairment as a clinical comorbidity in patients with epilepsy, we assessed the impact of BROC on cognition using three behavioral tests: shuttle box test and IntelliCage learning test. The novel object recognition test is designed to evaluate exploration, memory, and object recognition capabilities in mice. When the recognition index was calculated, either based on distance (Figure 2A) or time spent exploring objects (Figure 2B), epileptic mice demonstrated a reduced recognition index compared to control mice (*p* < 0.0001). Conversely, BROC‐treated epileptic mice exhibited significantly enhanced recognition indexes compared to untreated epileptic mice (*p* < 0.0001).

**FIGURE 3:**
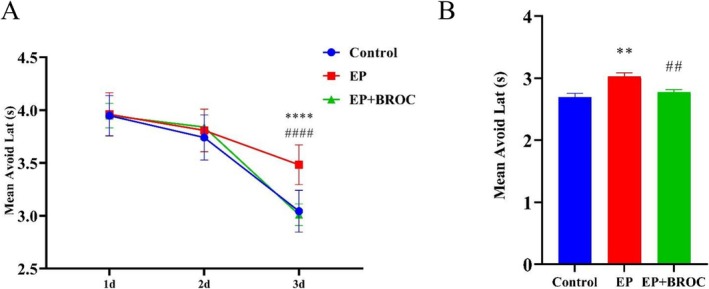
Analysis of mice behavior in shuttle box test. (A) Escape latency in the training phase. (B) Escape latency in the test phase. (*n* = 10; mean ± SEM; Repeated‐measures ANOVA followed by SNK multiple comparison test; **p < 0.01, ^****^ *p* < 0.0001 vs. Control group, ^##^ *p* < 0.01, ^####^ *p* < 0.0001 vs. EP group).

The shuttle box test is utilized to assess learning and memory in rodents, leveraging their fear response. Epileptic mice (EP group) demonstrated longer avoidance latencies compared to control mice or broccoli‐treated epileptic mice (EP + BROC group) (*p* < 0.0001). Meanwhile, there was no significant difference in avoidance latencies between control mice and mice in the EP + BROC group (Figure 3A). Furthermore, compared to control mice, epileptic mice showed a significantly increased mean avoidance latency in the EP group (*p* < 0.01), while BROC‐treated epileptic mice exhibited a reduced mean avoidance latency compared to untreated epileptic mice (*p* < 0.01) (Figure 3B).

**FIGURE 4:**
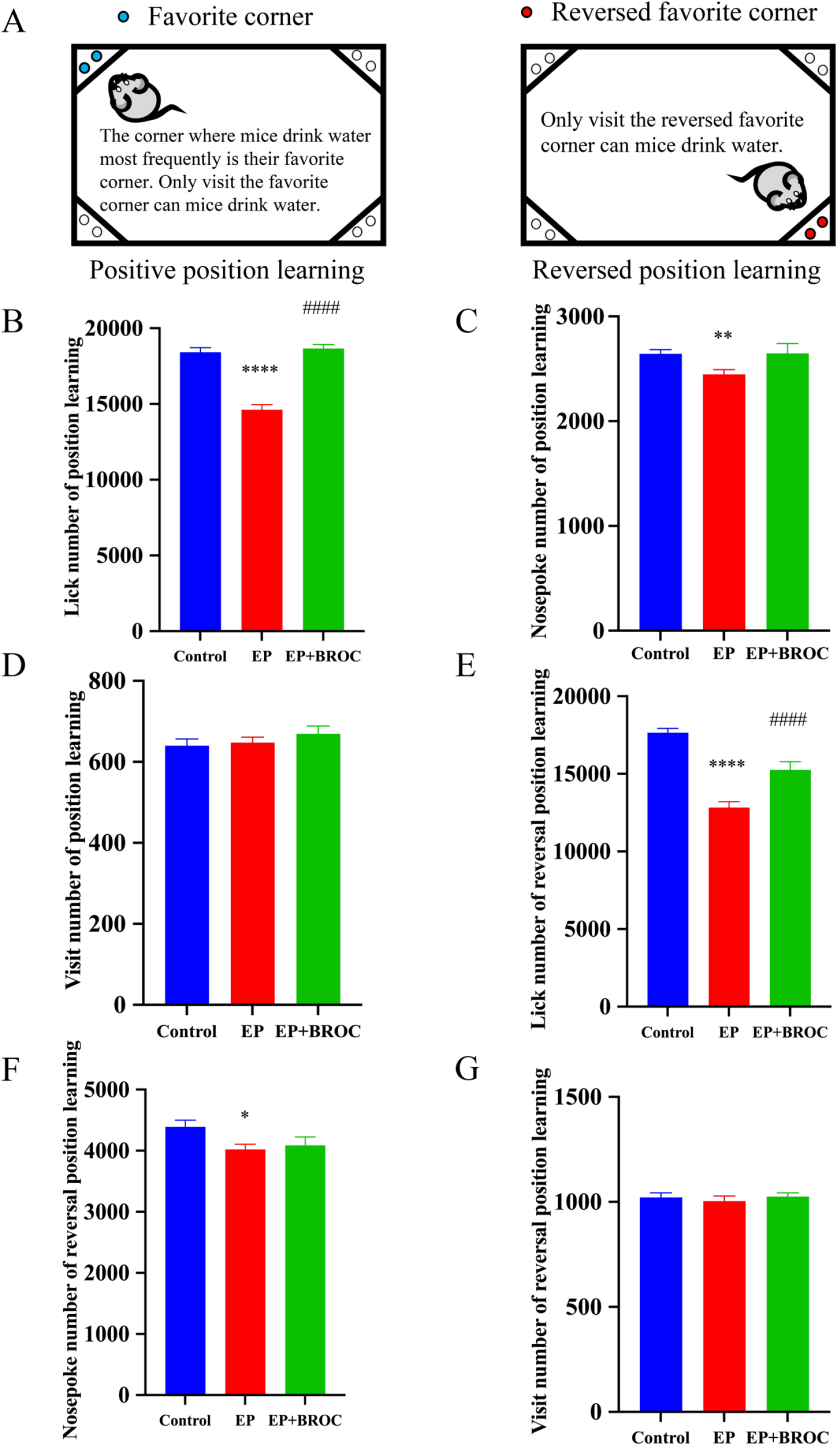
Analysis of mice behavior in IntelliCage learning test. (A) Architectural diagram and experimental procedure settings of the IntelliCage. The proportions of the cages are not drawn to scale. (B) Lick number during the position learning phase. (C) Nosepoke number during the position learning phase. (D) Visit number during the position learning phase. (E) Lick number during the reversal position learning phase. (F) Nosepoke number during the reversal position learning phase. (G) Visit number during reversal position learning phase. (*n* = 10; mean ± SEM; One‐way ANOVA followed by SNK multiple comparison test; *p < 0.05, **p < 0.01, ^****^ *p* < 0.0001 vs. Control group, ^####^ *p* < 0.0001 vs. EP group).

The IntelliCage system assesses cognitive ability in socially housed mice. During the position learning phase, compared to control mice, epileptic mice showed a decrease in lick numbers (*p* < 0.0001) and nosepoke numbers (*p* < 0.01), while BROC‐treated epileptic mice experienced an increase in lick numbers relative to untreated epileptic mice (*p* < 0.0001) (Figure 4B,C). No significant difference in visit numbers was observed among the groups (*p* > 0.05) (Figure 4D). During the reversal position learning phase, epileptic mice again exhibited reduced lick numbers (*p* < 0.0001) and nosepoke numbers (*p* < 0.05) compared to control mice (Figure 4E,F). Additionally, BROC treatment led to an increase in lick numbers in epileptic mice (*p* < 0.0001) (Figure 4E).

### 3.3 Freeze‐Dried Broccoli Powder May Promote MDA and Anti‐Oxidative Enzyme Levels in the Hippocampus of Epileptic Mice

**FIGURE 5:**
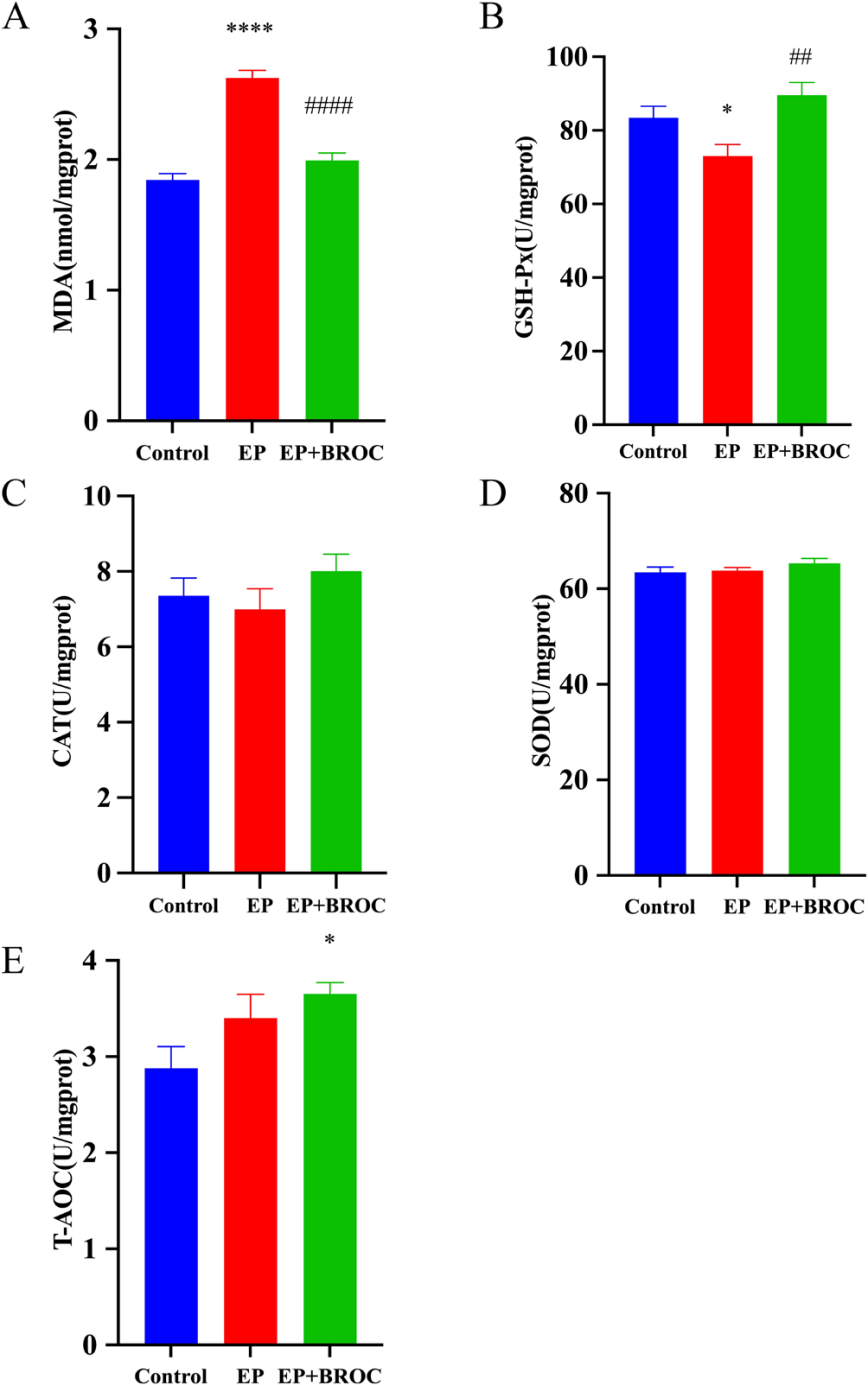
Analysis of MDA and anti‐oxidative enzyme levels in the hippocampus of mice among groups. (A) Contents of MDA. (B) Activities of GSH‐Px. (C) Activities of CAT. (D) Activities of SOD. (E) Activities of T‐AOC. (*n* = 10; mean ± SEM; One‐way ANOVA followed by SNK multiple comparison test; *p < 0.05, ^****^ *p* < 0.0001 vs. Control group, ^##^ *p* < 0.01, ^####^ *p* < 0.0001 vs. EP group).

MDA, a stable end product of lipid peroxidation, serves as a biomarker for assessing OS in various biological samples. GSH‐Px, CAT, SOD, and T‐AOC represent crucial antioxidant enzymes, indicative of the body's antioxidant status. As shown in Figure 5A,B, in comparison to the control group, a significant elevation in MDA levels (*p* < 0.0001) and a marked reduction in GSH‐Px activity (*p* < 0.05) were observed in the hippocampus of epileptic mice. Conversely, relative to untreated epileptic mice, BROC treatment led to a notable decrease in MDA levels (*p* < 0.0001) (Figure 5A), a significant increase in GSH‐Px activity (*p* < 0.01) (Figure 5B), and an enhancement of T‐AOC activity (*p* < 0.05) (Figure 5E) in the hippocampus. However, no significant changes in CAT and SOD activities were detected among the groups (*p* > 0.05) (Figure 5C,D).

### 3.4 Freeze‐Dried Broccoli Powder May Promote the Activation of Transcription Factor Nrf2 in the Hippocampus of Epileptic Mice

**FIGURE 6:**
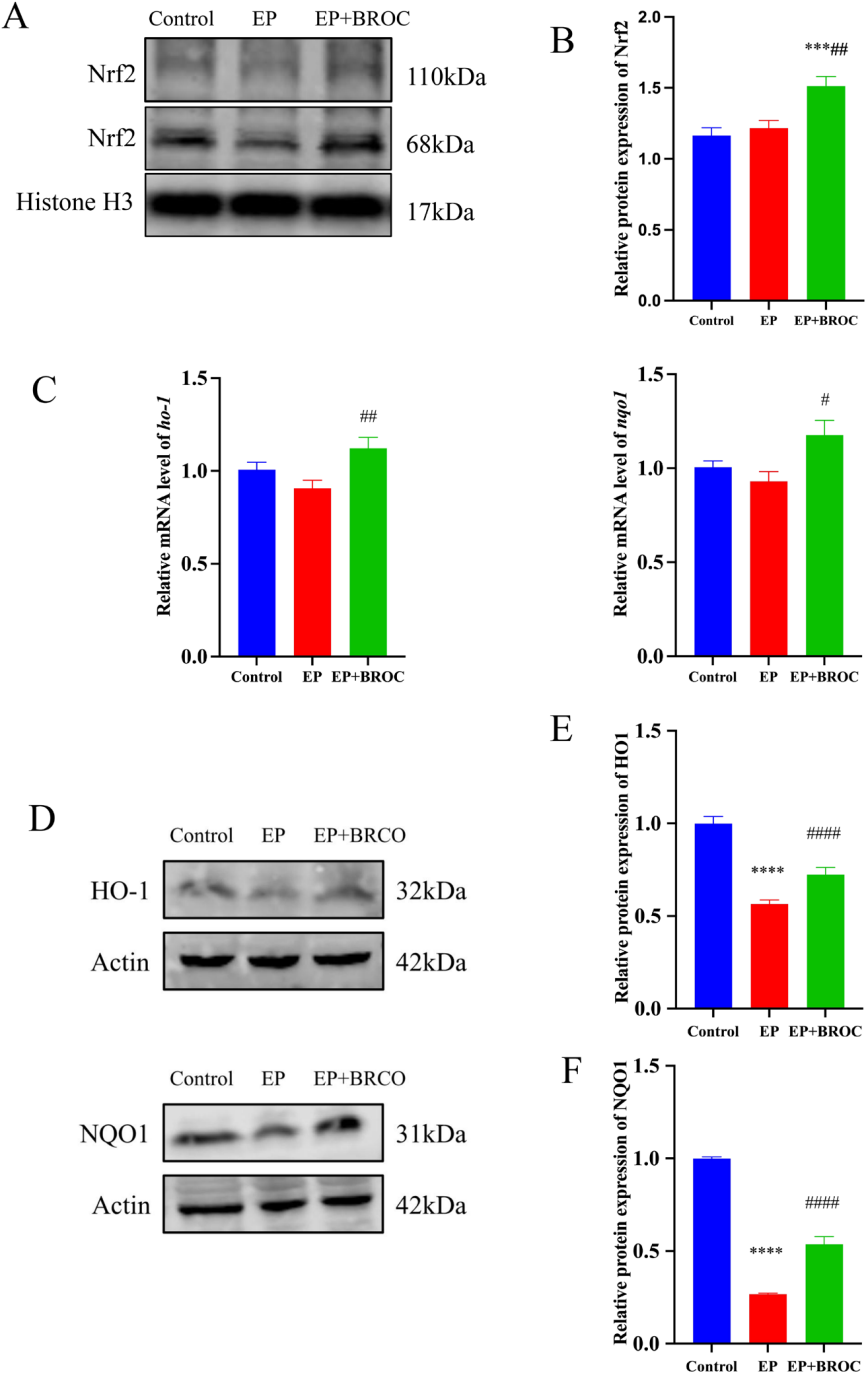
Analysis of Nrf2 levels in nuclear fraction, HO‐1, and NQO1 in total proteins from hippocampus among groups. (A,B) Nrf2 levels in the nuclear fraction in the hippocampus. (C) Relative transcription levels of *ho‐1* and *nqo1*. (D–F) Relative expression levels of HO‐1 and NQO1. (*n* = 10; mean ± SEM; One‐way ANOVA followed by SNK multiple comparison test; **p < 0.01, ^****^ *p* < 0.0001 vs. Control group, ^#^ *p* < 0.05, ^##^ *p* < 0.01, ^####^ *p* < 0.0001 vs. EP group).

We examined the expression levels of nuclear transcription factor Nrf2, as well as the transcription and translation levels of two Nrf2‐regulated enzymes, HO‐1 and NQO1, in the hippocampus. As depicted in Figure 6A,B, BROC treatment elevated Nrf2 levels in the hippocampus of epileptic mice (*p* < 0.01), while no significant difference was observed between epileptic mice and control mice (*p* > 0.05). Additionally, BROC treatment enhanced the transcription levels of HO‐1 and NQO1 compared to untreated epileptic mice (*p* < 0.01 and *p* < 0.05, respectively) (Figure 6C). Compared to the control group, the expression levels of HO‐1 and NQO1 were significantly reduced in the EP group (*p* < 0.0001). BROC treatment substantially enhanced the expression levels of these two enzymes in untreated epileptic mice (*p* < 0.0001) (Figure 6E,F).

## 4 Discussion

Temporal lobe epilepsy (TLE) is the most common form of refractory epilepsy. Clinical studies have identified that TLE patients often experience cognitive impairment, as the persistent and recurrent seizures lead to chronic dysfunction of the central nervous system (Li et al. 2023). Importantly, the present study emphasizes that the potential link between the generation of reactive oxygen species (ROS) during seizures and subsequent oxidative stress (OS) has been significantly associated with neuroinflammation (Yakimov et al. 2023) and post‐epilepsy cognitive dysfunction. Cruciferous vegetables, such as broccoli, are recognized for their antioxidant and anti‐inflammatory properties (Treasure et al. 2023), and studies have demonstrated that broccoli powder supplementation can improve cognitive function in both elderly and adolescent populations (Liu et al. 2024; Shirai et al. 2015). As a functional food, broccoli contains various bioactive compounds, and freeze‐drying has been shown to best preserve its antioxidant activity among different processing methods (Xu et al. 2020). Therefore, we administered 4% freeze‐dried broccoli powder mixed into feed to epileptic mice for 88 days, which was expected to ensure optimal antioxidant efficacy while avoiding potential toxicity (Canene‐Adams et al. 2013; Ma et al. 2022; Tomofuji et al. 2012). We presented evidence that underscores the beneficial effects of freeze‐dried broccoli powder on cognitive impairment in a lithium‐pilocarpine‐induced epileptic mouse model. Specifically, our findings reveal that this treatment boosts the antioxidant capacity of the hippocampus and exhibits significant neuroprotective effects, suggesting the potential of BROC as an adjunctive therapeutic agent for post‐epileptic cognitive dysfunction.

The chemically induced lithium‐pilocarpine status epilepticus animal model is a reliable and cost‐effective method (Che Has 2023). Pilocarpine can activate Cholinergic Receptor, Muscarinic 1 (CHRM1), leading to hippocampal NMDA receptor activation and the enhanced excitation of glutamatergic neurons (Niquet et al. 2023). The systemic application of pilocarpine in rats selectively induces epileptic activity in limbic system structures, accompanied by occasional motor seizures, transient epileptic status, and widespread brain damage, closely mirroring persistent status epilepticus in humans (Turski et al. 1983). Furthermore, numerous studies have demonstrated that lithium‐pilocarpine‐induced epileptic rodents exhibit various behavioral alterations and impaired memory function (Smolensky et al. 2019; Zubareva et al. 2023). We employed the IntelliCage system, the novel object recognition test, and the shuttle box test to assess model validity and cognitive performance. The IntellCage learning tests represent a high‐throughput system capable of simultaneously assessing the basic behavioral navigation and a diverse array of complex behavioral and cognitive functions in rodents (Iman et al. 2021). Novel object recognition, known for its relatively low‐stress nature, serves as an effective memory test for mice, suitable for identifying neurobehavioral alterations following pharmacological, biological, or genetic manipulations (Lueptow 2017). Additionally, the shuttle box test stands as the most utilized behavioral assessment tool for gauging the learning and memory capabilities of rodents (Berezhnoy et al. 2020). Indeed, consistent with previous findings (Wang et al. 2021; Yang et al. 2023), our epileptic mice showed significant cognitive deficits and impairments in learning and memory. In behavioral assessments compared to control mice, epileptic mice exhibited significant reductions in cognitive functions, alongside notable impairments in learning and memory. Our results demonstrated that BROC treatment effectively improved cognitive function in epileptic mice.

Studies have shown that epilepsy can trigger the expression of reactive oxygen species (ROS) and induce OS, which can ultimately lead to the death of neuronal cells (Shi et al. 2018). To address this issue, we focused on BROC, which contains various bioactive compounds, including phenolic compounds, sulfur‐containing compounds, carotenoids, and tocopherols, all of which demonstrate significant biological activities (Ramirez et al. 2020). Among these, flavonoids from phenolic compounds improve antioxidant status by regulating glutathione levels, enhancing antioxidant enzyme activities, and scavenging free radicals (Kobori et al. 2015; Zhang et al. 2023b). In addition to phenolic compounds, Glucoraphanin is hydrolyzed by myrosinase to form isothiocyanates, among which sulforaphane (SFN) is the primary active component. SFN protects neurons and microglia by reducing OS and inflammation through activation of the Nrf2 pathway (Kraft et al. 2004; Ladak et al. 2021). Studies have shown that SFN can improve cognitive dysfunction in various models (Hua et al. 2022; Rajesh and Ilanthalir 2016). Additionally, carotenoids and tocopherols in broccoli exhibit antioxidant and anti‐inflammatory properties through synergistic effects (Di Vincenzo et al. 2019; Stahl and Sies 2003). In mice with epilepsy, treatment with SFN resulted in a notable elevation in the levels of Nrf2, NQO1, and HO‐1 in brain tissue (Sandouka and Shekh‐Ahmad 2021). Consequently, the increased expression of Nrf2 enhances the activity of the endogenous antioxidant system, improves the functions of enzymes such as SOD and GSH‐Px, reduces MDA levels, and limits the excessive accumulation of ROS. These changes are crucial in decreasing neuronal loss and slowing the progression of epilepsy (Shekh‐Ahmad et al. 2019; Wu et al. 2020). In our study, treatment with BROC was observed to significantly elevate the concentration of MDA and the activities of both GSH‐Px and T‐AOC in epileptic murine hippocampal tissues. In epilepsy, an increase in T‐AOC signifies the mobilization of endogenous pathways designed to counteract oxidative stress. Our BROC intervention substantially enhanced T‐AOC levels, indicating its potent antioxidative effects. This enhancement significantly attenuates oxidative stress, thereby ameliorating the associated neurological damage in epilepsy. Furthermore, it also augmented the nuclear protein level of Nrf2 and the transcription and translation levels of HO‐1 and NQO1. Collectively, these results indicated that BROC's neuroprotective effects might be mediated through enhancement of antioxidant defense, particularly via the Nrf2 pathway.

## 5 Conclusions and Limitations

Our study demonstrates that BROC significantly attenuates cognitive and memory impairments in epileptic mice and enhances hippocampal tissue antioxidant capacity with neuroprotective effects. These findings indicate the therapeutic potential of BROC intervention for post‐epileptic cognitive dysfunction. Despite these promising results, several limitations should be noted. Primarily, our investigation was limited to evaluating BROC's overall efficacy in the absence of quantitative analysis of its specific bioactive components, which precludes comprehensive elucidation of the underlying mechanisms. Additionally, given that cognitive dysfunction following epilepsy is a time‐dependent process, our study investigated only short‐term outcomes, potentially limiting the temporal scope of our conclusions regarding cognitive changes and chronic effects. Moreover, as this research remains at the preclinical stage, the translational applicability of our findings requires further investigation. In the future, research should be directed towards delineating the molecular mechanisms of BROC's bioactive constituents and conducting comprehensive longitudinal clinical studies.

## Author Contributions

**Yanan Gong:** conceptualization (equal), data curation (equal), formal analysis (equal), investigation (equal), writing – original draft (equal). **Hongzhen Zhou:** conceptualization (equal), data curation (equal), formal analysis (equal), investigation (equal), writing – original draft (equal). **Xinle She:** formal analysis (equal), investigation (equal), writing – review and editing (equal). **Yan Guo:** conceptualization (equal), data curation (equal), formal analysis (equal), investigation (equal), writing – original draft (equal). **Yongzhong Zhou:** formal analysis (equal). **Nan Peng:** data curation (equal). **Guoli Zhou:** formal analysis (equal). **Tengwei Gao:** investigation (equal). **Furong Liu:** investigation (equal). **Yiqian Wang:** investigation (equal). **Jing Ye:** investigation (equal). **Jing Jin:** writing – review and editing (equal). **Rui Zhang:** supervision (equal), writing – review and editing (equal).

## Ethics Statement

This study was approved by the Animal Care and Use Committee of Ningxia Medical University (Ethical Approval No. NXMU2020‐021).

## Conflicts of Interest

The authors declare no conflicts of interest.

## Supporting information


Data S1.

## Data Availability

The data that support the findings of this study are available from the corresponding author upon reasonable request.
