# Supplementary material for: Unlocking the Potential of Freeze‐Dried Broccoli Powder: A Novel Approach to Enhancing Cognitive Resilience in Temporal Lobe Epilepsy
Source: Food Sci Nutr. 2025 Mar 8;13(3):e70079. doi: 10.1002/fsn3.70079 (PMC11889413; doi:10.1002/fsn3.70079)
Supplement: Supplementary file 1 — Data S1. [file FSN3-13-e70079-s001.pptx]

## Slide 1
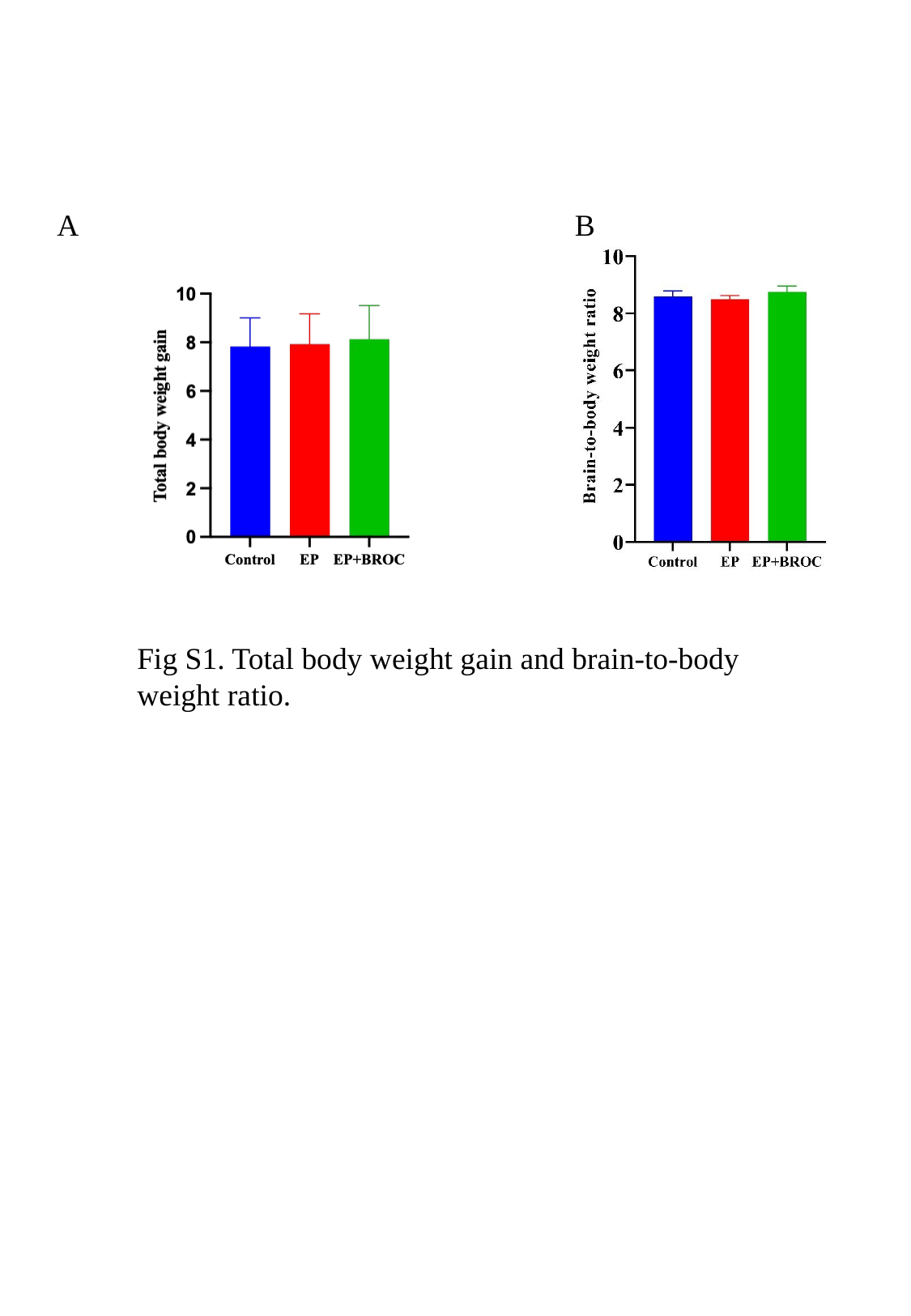

A
B
Fig S1. Total body weight gain and brain-to-body weight ratio.

## Slide 2
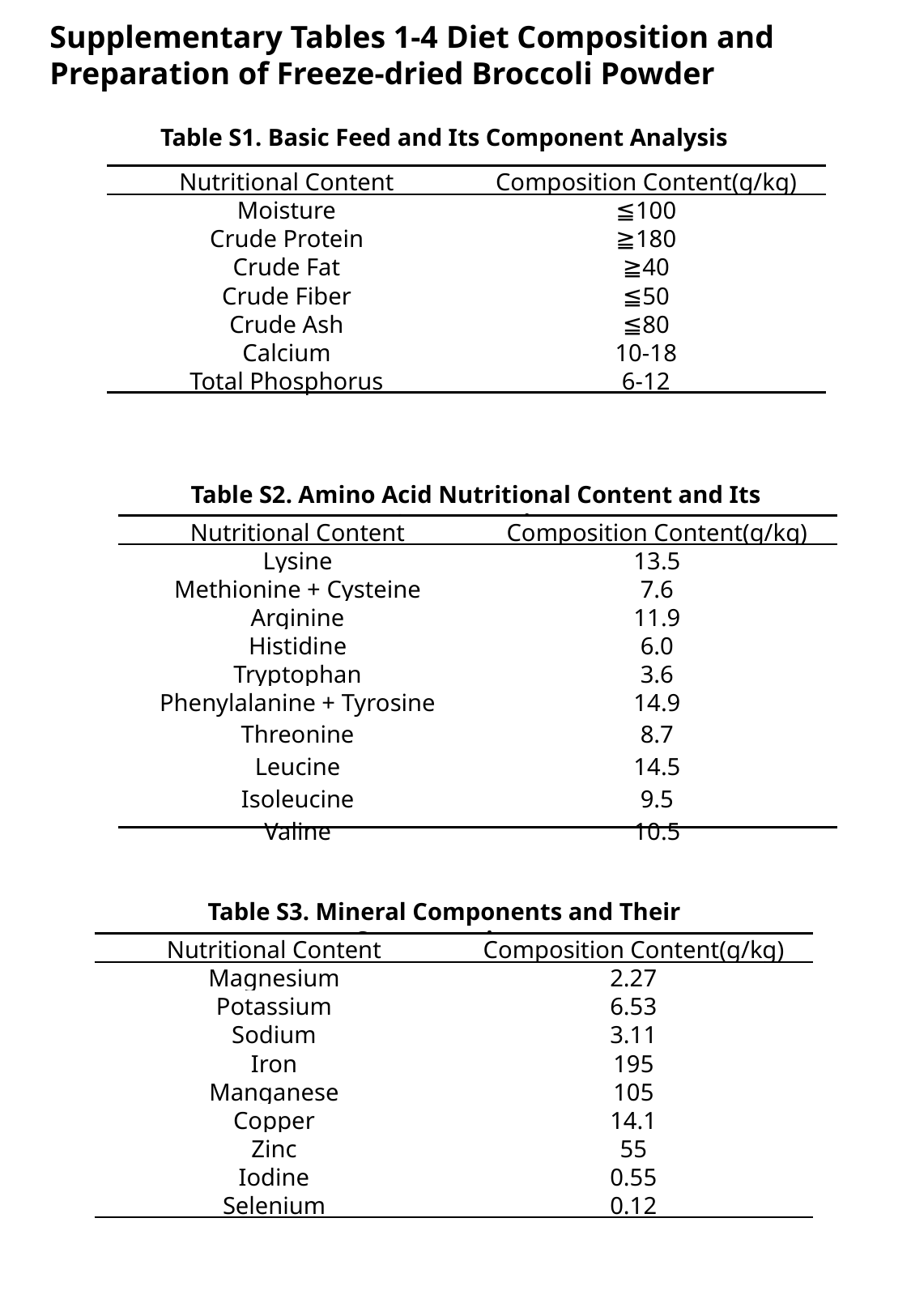

Supplementary Tables 1-4 Diet Composition and Preparation of Freeze-dried Broccoli Powder
Table S1. Basic Feed and Its Component Analysis
| Nutritional Content | Composition Content(g/kg) |
| --- | --- |
| Moisture | ≦100 |
| Crude Protein | ≧180 |
| Crude Fat | ≧40 |
| Crude Fiber | ≦50 |
| Crude Ash | ≦80 |
| Calcium | 10-18 |
| Total Phosphorus | 6-12 |
Table S2. Amino Acid Nutritional Content and Its Concentration
| Nutritional Content | Composition Content(g/kg) |
| --- | --- |
| Lysine | 13.5 |
| Methionine + Cysteine | 7.6 |
| Arginine | 11.9 |
| Histidine | 6.0 |
| Tryptophan | 3.6 |
| Phenylalanine + Tyrosine Threonine Leucine Isoleucine Valine | 14.9 8.7 14.5 9.5 10.5 |
Table S3. Mineral Components and Their Concentrations
| Nutritional Content | Composition Content(g/kg) |
| --- | --- |
| Magnesium | 2.27 |
| Potassium | 6.53 |
| Sodium | 3.11 |
| Iron | 195 |
| Manganese | 105 |
| Copper | 14.1 |
| Zinc | 55 |
| Iodine | 0.55 |
| Selenium | 0.12 |

## Slide 3
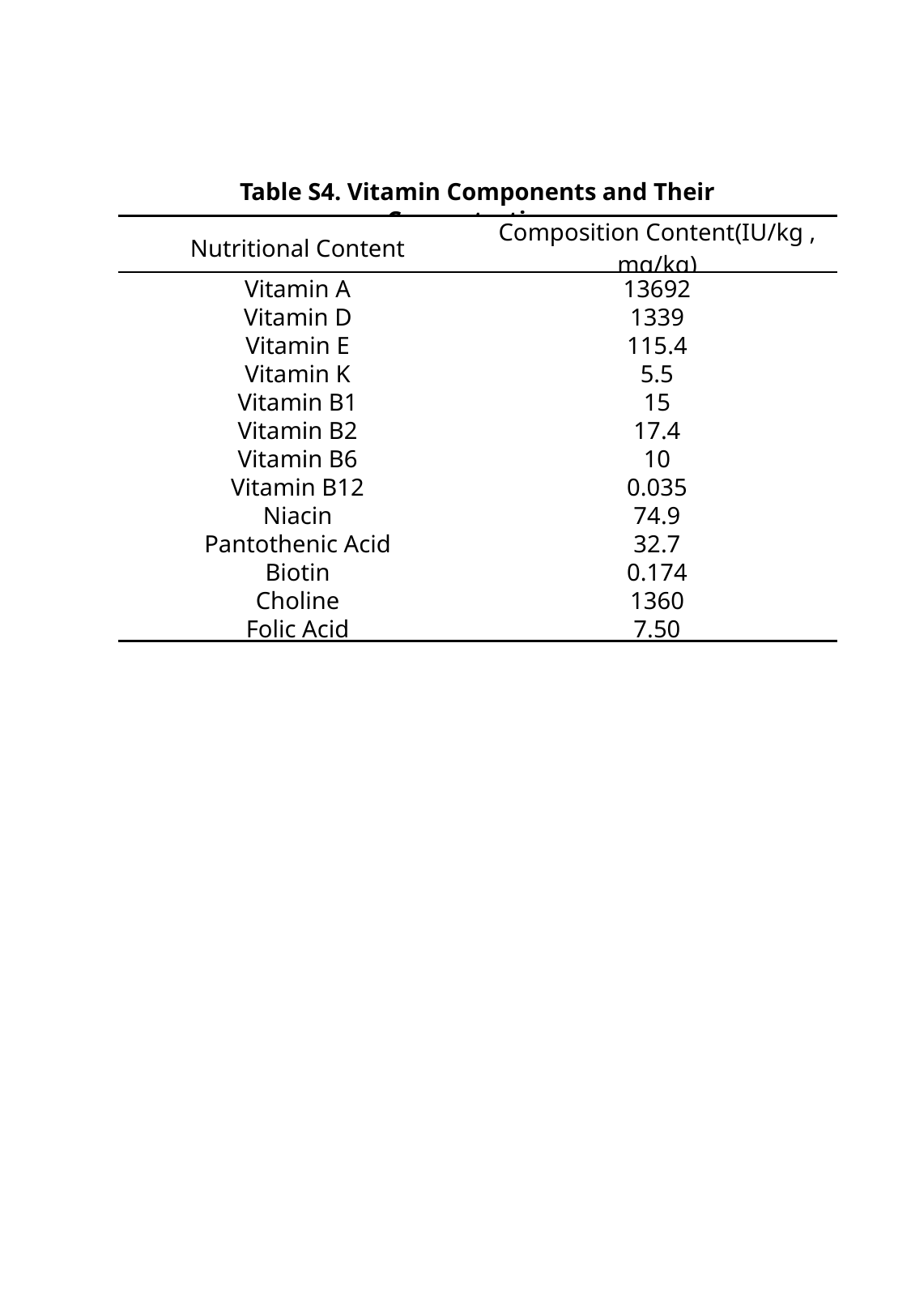

Table S4. Vitamin Components and Their Concentrations
| Nutritional Content | Composition Content(IU/kg , mg/kg) |
| --- | --- |
| Vitamin A | 13692 |
| Vitamin D | 1339 |
| Vitamin E | 115.4 |
| Vitamin K | 5.5 |
| Vitamin B1 | 15 |
| Vitamin B2 | 17.4 |
| Vitamin B6 | 10 |
| Vitamin B12 | 0.035 |
| Niacin | 74.9 |
| Pantothenic Acid | 32.7 |
| Biotin | 0.174 |
| Choline | 1360 |
| Folic Acid | 7.50 |
